# Tuning the Magnetic Properties of Nanoparticles

**DOI:** 10.3390/ijms140815977

**Published:** 2013-07-31

**Authors:** Arati G. Kolhatkar, Andrew C. Jamison, Dmitri Litvinov, Richard C. Willson, T. Randall Lee

**Affiliations:** 1 Department of Chemistry and Texas Center for Superconductivity, University of Houston, Houston, TX 77204-5003, USA; E-Mails: akolhatkar@uh.edu (A.G.K.); ajamison@uh.edu (A.C.J.); 2 Department of Chemical and Biomolecular Engineering, University of Houston, Houston, TX 77204-5003, USA; 3 Department of Electrical and Computer Engineering, University of Houston, Houston, TX 77004-4005, USA

**Keywords:** modulating, magnetization, coercivity, relaxation, magnetic properties, size, shape, composition, shell-core, nanoparticle

## Abstract

The tremendous interest in magnetic nanoparticles (MNPs) is reflected in published research that ranges from novel methods of synthesis of unique nanoparticle shapes and composite structures to a large number of MNP characterization techniques, and finally to their use in many biomedical and nanotechnology-based applications. The knowledge gained from this vast body of research can be made more useful if we organize the associated results to correlate key magnetic properties with the parameters that influence them. Tuning these properties of MNPs will allow us to tailor nanoparticles for specific applications, thus increasing their effectiveness. The complex magnetic behavior exhibited by MNPs is governed by many factors; these factors can either improve or adversely affect the desired magnetic properties. In this report, we have outlined a matrix of parameters that can be varied to tune the magnetic properties of nanoparticles. For practical utility, this review focuses on the effect of size, shape, composition, and shell-core structure on saturation magnetization, coercivity, blocking temperature, and relaxation time.

## 1. Introduction

Magnetic nanoparticles (MNPs) have been extensively studied over the last half century and continue to sustain interest due to their potential use in fields ranging from high-density data storage [1] to biomedical applications [2,3]. The unique properties of MNPs derive from the fact that these nanoscale magnets differ from bulk materials due to their high surface-to-volume ratios. Owing to strong interest in their possible applications, several reviews of MNPs have been published [2,4], including those that focus on sensing [1,5], drug delivery [6–8], and hyperthermia [9]. Although there is a plethora of published information, a review that emphasizes the optimization of MNP properties to effectively target specific applications is lacking. The motivation for assembling this report was to provide a matrix of parameters to modulate and tune the properties of MNPs for a particular end-use. Recently, there has been substantial progress in the synthesis of MNPs of varying sizes, shapes, compositions, and shell-core designs [10,11]. This review will target the different factors that contribute to the control and optimization of the key magnetic properties of MNPs: saturation magnetization (Ms), coercivity (Hc), blocking temperature (T_B_), and relaxation time (t_N_ and t_B_).

MNPs have already been utilized in several biomedical applications [6,7,12–14]. To demonstrate how MNP structure and the resulting properties are intertwined, we can use a specific application to identify the parameters that tune crucial magnetic properties. In biosensing, for example, nanoparticles with higher saturation magnetization are preferred because they provide higher sensitivity and efficiency [2]. It has been demonstrated in several studies (*vide infra*) [15] that saturation magnetization increases linearly with size until it reaches the bulk value. While the correlation between magnetization and shape is not as direct, the effect of geometry on magnetic properties continues to be evaluated for biosensing applications [16,17]. A recent report pointed out the increased sensitivity of cubic MNPs for a biosensing platform owing to the increase in contact area for a cube in comparison to a sphere [18]. Composition also plays a significant role in influencing magnetic properties. However, due to concerns about the toxicity of the elements or compounds involved, the effect of the variation of composition has generally only been examined for *ex vivo* applications; consequently, data related to applications involving biological contact reflect these limitations. For implantable biosensors such as glucose monitoring systems, biocompatibility has been a significant challenge. These concerns also exist for the various magnetic materials used in research and have frequently been addressed by encapsulating the MNP in an appropriate coating [19]. The nature of the coating is an important consideration in such shell-core MNP designs since the coating might enhance or significantly reduce the magnetic properties of the core based on the interaction between the ligand and the nanoparticle surface [7], the relative thickness of the shell, and the size of the nanoparticle being coated [20,21].

**Figure 1:**
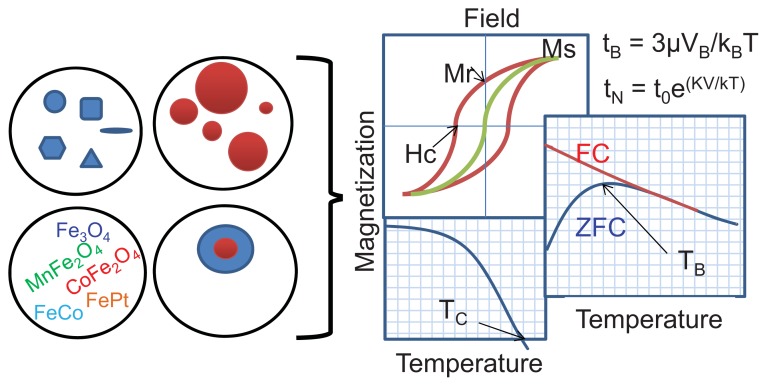
Effects of various parameters (e.g., shape, size, composition, architecture) on the magnetic properties of MNPs. (Abbreviations and magnetic property-based nomenclature has been defined and discussed in the following sections).

From this initial example, it is apparent that an understanding of the effectiveness of the various types of MNPs from a specific application-based perspective fails to provide the full picture of how to optimize an MNP system. For this reason, the bulk of the text that follows will focus on the influence of specific parameters on magnetic properties. Although we are aware that a combination of parameters might be involved in determining the effectiveness of a MNP for a specific application, for simplicity, we have listed tunable magnetic properties of fundamental importance for several applications in Table 1. These properties will be defined in the following section. We have also provided a brief list of published research focused on the key MNP parameters in Table 2. To maintain the practical utility of this review, we have focused on the following parameters that can be easily manipulated to tune the magnetic properties of the MNPs (Figure 1) using appropriate synthesis methods: (1) size; (2) shape; (3) composition; and (4) shell-core design. However, to provide context, the section that follows briefly outlines the fundamentals of nanomagnetism.

## 2. Nanomagnetism

The design of MNPs with tailored properties depends on the fundamental concepts of nanomagnetism (*i.e.*, magnetism observed in nanoparticles). A review of what produces magnetization, including the relationship between various extrinsic and intrinsic parameters, will enable us to better evaluate the underlying factors that influence magnetism at the nanoscale. Explanations about the role of atomic and molecular structure upon magnetization are readily available [55]. However, from a practical perspective, most of what we need to know to manipulate the effectiveness of these nanoscale magnets can be derived from prior experimental observations and an understanding of the role of MNP magnetic domain structure.

**Figure 2:**
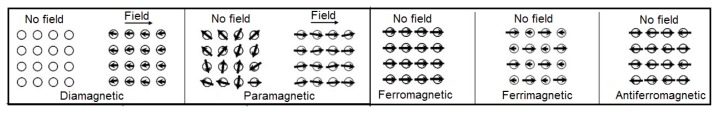
Magnetic dipoles and behavior in the presence and absence of an external magnetic field. Based on the alignment and response of magnetic dipoles, materials are classified as diamagnetic, paramagnetic, ferromagnetic, ferrimagnetic, antiferromagnetic. Reproduced with permission from [57].

Based on the response of the intrinsic MNP magnetic dipole and the net magnetization in the presence and absence of an applied magnetic field, MNPs are typically classified as being either diamagnetic, paramagnetic, ferromagnetic, ferrimagnetic, and antiferromagnetic [56,57]. Figure 2 shows the net magnetic dipole arrangement for each of these types of magnetic materials. For diamagnetic materials in the absence of a magnetic field, magnetic dipoles are not present. However, upon application of a field, the material produces a magnetic dipole that is oriented opposite to that of the applied field; thus, a material that has strong diamagnetic character is repelled by a magnetic field. For paramagnetic materials, there exist magnetic dipoles as illustrated in Figure 2, but these dipoles are aligned only upon application of an external magnetic field. For the balance of the magnetic properties illustrated in Figure 2, the magnetization in the absence of an applied field reveals their fundamental character. Ferromagnetic materials have net magnetic dipole moments in the absence of an external magnetic field. In antiferromagnetic and ferrimagnetic materials, the atomic level magnetic dipole moments are similar to those of ferromagnetic materials, however, adjacent dipole moments exist that are not oriented in parallel and effectively cancel or reduce, respectively, the impact of neighboring magnetic dipoles within the material in the absence of an applied field.

Research in magnetic nanoparticles typically focuses on developing an optimal response for MNPs to an external magnetic field, and the majority of the published research has involved MNPs that are typically classified as either ferrimagnetic, ferromagnetic, or superparamagnetic particles (a special case of ferro- or ferri-magnetic particles). Below certain critical dimensions (that vary with the material parameters), MNPs exhibit magnetic responses reminiscent of those of paramagnetic materials, which is a zero average magnetic moment in the absence of an external field and a rapidly increasing (as compared to paramagnetic materials) magnetic moment under application of an external field in the direction of the field. This phenomenon, observed at temperatures above the so-called blocking temperature (see below), arises from the thermal fluctuations within the nanoparticles being comparable to or greater than the energy barrier for moment reversal, allowing rapid random flipping of the nanoparticle magnetic moments. In the case where the magnetization of the MNP over the measurement/observation interval is equal to zero in the absence of an external field, such nanoparticles are referred to as *superparamagnetic*. Superparamagnetism is especially important in applications such as drug delivery or MRI, where the nanoparticles exhibit no magnetic properties upon removal of the external field and therefore have no attraction for each other, eliminating the major driving force for aggregation. More importantly, superparamagnetic nanoparticles allow better control over the application of their magnetic properties because they provide a strong response to an external magnetic field.

**Figure 3:**
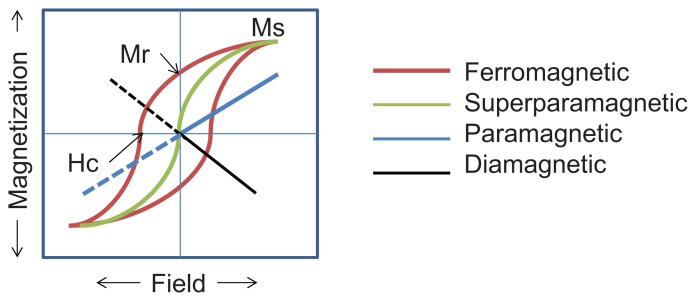
Magnetic behavior under the influence of an applied field, as further described in the text. The X-axis is the applied field (Oe), and the Y-axis is the magnetization of the sample as a function of field exposure (emu/g). Reproduced with permission from [6].

For MNPs, the maximum magnetization possible is called the saturation magnetization, and it arises when all the magnetic dipoles are aligned in an external magnetic field. Figure 3 shows a typical magnetization curve for ferromagnetic or ferrimagnetic nanoparticles showing the characteristic positions on the curve associated with *saturation magnetization* (Ms, maximum induced magnetization), *remanent magnetization* (Mr, induced magnetization remaining after an applied field is removed), and *coercivity* (H, the intensity of an external coercive field needed to force the magnetization to zero). In the same figure, in contrast to the hysteresis observed in the case of ferromagnetic nanoparticles (red loop), the response of superparamagnetic nanoparticles to an external field also follows a sigmoidal curve but shows no hysteresis (green line). The response of paramagnetic (blue line) and diamagnetic (black line) nanoparticles is also shown in the schematic. The Ms shown in Figure 3 depends on temperature and is at a maximum at 0 K when the thermal vibrations (and thus randomization of aligned moments) are reduced.

**Figure 4:**
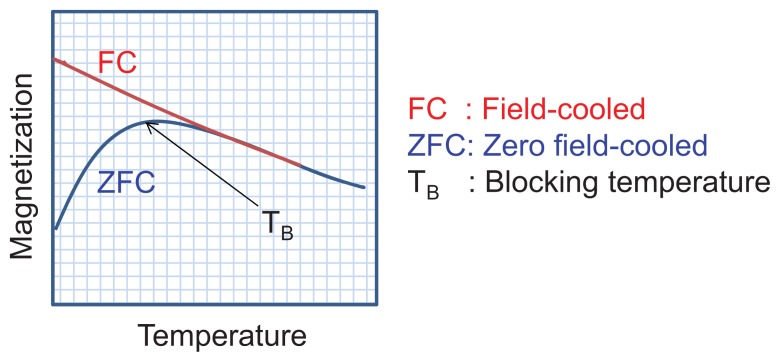
Experimental strategy for estimating the blocking temperature of magnetic nanoparticles.

Above the temperature known as the blocking temperature (T_B_), both ferromagnetic and ferrimagnetic nanoparticles exhibit superparamagnetic behavior manifested by rapid random MNP magnetization reversals leading to a zero time-average magnetic moment. The value of T_B,_ associated with the energy barrier, depends on the characteristic measuring time, which can vary from 100 to 10^−8^ s [58]. The magnetic behavior arises from the relative difference between the measuring time and the relaxation time. If the measuring time is greater than the relaxation time, the nanoparticles are considered to be in the superparagmagnetic regime; if, however, the measuring time is less than the relaxation time, the nanoparticles are in a “blocked” (ferromagnetic) regime [58]. Experimentally, the value of T_B_ typically corresponds to the “merging point” of the zero-field cooled (ZFC) and field-cooled (FC) magnetization curves [59]. In ZFC measurements, a sample is first cooled to low temperature (e.g., 2–10 K) in the absence of an external field (zero-field). At this point, a small external field is applied, and the temperature is gradually increased while measuring the sample magnetization as a function of temperature. In FC measurements, the process is repeated, but the sample is cooled in the presence of an external field (~50 Oe) and the same external field is applied as the temperature is increased. As shown in Figure 4, the point where the two curves merge is the irreversibility temperature, T_irr_, and the maximum on the ZFC curve is the blocking temperature, T_B_ (Figure 4).

**Figure 5:**
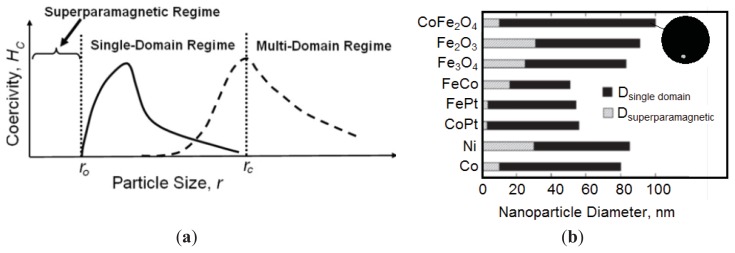
(**a**) Transition from superparamagnetic to single to multi-domain regimes. Reproduced with permission from [57]; (**b**) Maximum diameters for superparamagnetic and single-domain nanoparticles of different compositions. Reproduced with permission from [63].

The value of T_B_ can also be estimated using Equation (1) if the values of magnetic anisotropy and the MNP’s size are known, and the particles have a single magnetic domain structure [58]:

(1) $${\text{T}}_{\text{B}}=\text{KV}/25{\text{k}}_{\text{B}}=\text{K}(4\pi {{\text{r}}_{\text{o}}}^{3}/3)/25{\text{k}}_{\text{B}}$$

where k_B_ is the Boltzmann constant, K is an anisotropy constant, and V is the volume of one MNP. From Equation (1), we can see that blocking temperatures rapidly increase with particle size. However, this equation is not necessarily applicable for larger MNPs, where regions of uniform magnetization are separated by domain boundaries that develop during the process of MNP nucleation and growth [4]. If the MNP size is maintained below a critical volume/size during nanoparticle synthesis, the MNPs tend to develop as single magnetic domain structures, and at the smallest sizes, they exhibit superparamagnetic behavior under standard conditions. These size regimes are illustrated in Figure 5.

The critical size (r_c_), which corresponds to a transition from the single-domain to the multi-domain regime, is complex [60]. One definition indicates that this size is associated with the point where it is energetically favorable for the magnetic grain (or particle) to exist without a domain wall [61,62], which might be interpreted as a maximum size for such single-domained structures, as depicted in Figure 5a. However, with the broad array of magnetic materials in use in MNP research, it is challenging to define a discrete transition point for r_c_, and the term “pseudo single-domain” has been used for structures that fall in the overlap between nanoparticles that are well defined as being either single-domain or multi-domain structures [56]. A domain wall is a transition region between the different magnetic domains of uniform magnetization that develops when a magnetic material forms domains to minimize the magnetostatic energy; wall energy is the energy required to maintain this wall. When domains form, the magnetostatic energy decreases, and the wall energy and the magnetocrystalline anisotropy energy increase. For a nanoparticle to split into domains, its size should be greater than the thickness of the domain wall. Therefore, the domain wall thickness (and thus the critical size, r_c_) depends on three parameters: the exchange energy (which is the energy required to keep the spins parallel and is low in the case of a thick wall), magnetization, and anisotropy of the nanoparticle.

The transition point from superparamagnetic to single-domain to multi-domain for each type of MNP depends upon the size and/or geometry of the nanoparticles, as shown for MNP size in Figure 5a, and upon the intrinsic material parameters such as Ms and K, as is illustrated in Figure 5b for MNPs having different compositions [2]. From Equation (1), above, we can estimate the size at which spherical nanoparticles transition from superparamagnetic to single-domain character as shown in Equation (2):

(2) $${\text{r}}_{\text{o}}={\left(6{\text{k}}_{\text{B}}{\text{T}}_{\text{B}}/\text{K}\right)}^{1/3}$$

where r_o_ is transition point from superparamagnetic to single domain (also illustrated in Figure 5a), k_B_ is the Boltzmann constant, T_B_ is the blocking temperature, and K is an anisotropy constant.

Nanomagnetism, which is responsible for superparamagnetic behavior and/or single/multi- domain behavior, is a vast topic, and the above discussion is a good starting point. It is important to understand the fundamental magnetic properties and their interdependence to be able to optimize them for a particular application. Application-specific concepts (like specific absorption rate (SAR)/specific loss power (SLP) for hyperthermia, proton relaxation, and contrast-enhancing efficiency in MRI applications) are discussed in the following sections as needed to describe the influence of various parameters on the magnetic properties of nanoparticles.

## 3. Effect of Different Parameters on Magnetic Properties

Although there is a strong and relatively well-established dependence of magnetic properties on the size of the nanoparticles, magnetic behavior is complicated and cannot be defined with respect to one parameter. Peddis *et al.* described examples of anomalous behaviors (e.g., where large nanoparticles exhibit superparamagnetic behavior or lower coercivity than that found in smaller particles), which suggest that other factors also influence key magnetic properties [26]. In the subsection that follows, we review the important role of size upon the magnetic character of MNPs, and will follow this discussion with subsections describing how shape, composition, and shell-core design can be utilized as parameters to optimize magnetic properties.

### 3.1. Size

It has been shown that many of the unique magnetic properties of nanoparticles can be attributed to their high surface-to-volume ratio [1,3]. Ms varies with size until it reaches a threshold size beyond which magnetization is constant and is close to the bulk value. The linear dependence of Ms on size below this threshold has been demonstrated in several studies, and a number of examples are provided in Table 3. However, the tunable property of size is subtractive with respect to Ms and superparamagnetism: for example, when the size decreases, the nanoparticle moves toward superparamagnetism but may have a reduced Ms. Depending on the targeted application, we might choose to tailor the size of the nanoparticles to tune these parameters (e.g., to favor superparamagnetism or high Ms).

As illustrated in Figure 5a, the size of the nanoparticle helps define the nanoparticle regime and hence its magnetic behavior. As the size of the MNP decreases, the magnetic anisotropy energy per nanoparticle decreases. Magnetic anisotropy energy is the energy that keeps the magnetic moment in a particular orientation. At a characteristic size for each type of MNP, the anisotropy energy becomes equal to the thermal energy, which allows the random flipping of the magnetic moment [64]. The flipping occurs at sizes below r_o_, and the nanoparticle is then defined as being superparamagnetic. The magnitude of Ms also strongly depends on the size of the nanoparticle and is described by Equation (3) [65]. MNPs possess a disordered spin layer at their surfaces, and when the size of the nanoparticle is small (<5 nm), the ratio of disordered layer to the radius of the MNP is significant. Surface spin disorder thus leads to reduced Ms for smaller nanoparticles as defined here:

(3) $$\text{Ms}={\text{Ms}}_{\text{b}}{\left[(\text{r}-\text{d})/\text{r}\right]}^{3}$$

where r is the radius, d is the thickness of the MNP surface exhibiting disordered spins, and Ms_b_ is the bulk Ms. Recent studies have demonstrated that the surface functionalization of MNPs can reduce the level of surface spin disorder observed in small nanoparticles, thus increasing their measured Ms [66,67]. Guardia *et al*. compared the magnetic properties of iron oxide (Fe_3_O_4_) MNPs of diameters 6, 10, and 17 nm and observed that the Ms of each unexpectedly reached the bulk value. They attributed this decrease in surface spin disorder (and hence increased magnetization) to covalent bonding of oleic acid to the nanoparticles. However, Nagesha *et al.* observed no such phenomenon when they examined 10 nm Fe_3_O_4_ MNPs that were dopamine-stabilized and oleic acid-stabilized. The Ms and T_B_ increased from 38 emu/g Fe and 30 K for oleic acid functionalization to 60 emu/g Fe and 50 K for dopamine functionalization. The authors observed a significant improvement in magnetic properties after dopamine functionalization, but unlike the previous study, the Ms of the 10 nm oleic acid-functionalized nanoparticles was only a fraction of the bulk value.

Due to their facile synthesis and potential for use in biomedical applications, Fe_3_O_4_ MNPs are commonly the focus of studies that evaluate the effect of various nanoparticle parameters on magnetic properties [10,68]. However, there is also substantial research regarding other types of nanoparticles tailored for specific applications. In Table 3, we have summarized recent studies that evaluated the effect of size upon the magnetic properties of different types of MNPs. In most of the studies listed here, the value of Ms increases with size until it reaches a maximum that is close to the bulk magnetization value; this trend appears to be independent of the synthetic route. Three studies by independent research groups using distinct methods of synthesis effectively demonstrated this assertion for Fe_3_O_4_ nanoparticles [23,24,32]. Additionally, in most of the studies shown in Table 3, the coercivity follows a similar trend, but after reaching a maximum, the coercivity decreases with size. The latter phenomenon occurs because as the size of the MNPs increases, the nanoparticles become pseudo single-domain and then multi-domain structures in which the moment of each domain may not be oriented in the same direction. On application of a magnetic field, some of the non-parallel moments cancel (vector addition of forces), leading to a reduced level of coercive field (coercivity) required to force the magnetization to zero. Although Guardia *et al.* have reported one of the highest Ms values found in the literature, they did not provide an explanation for their sinusoidal trends for coercivity of the Fe_3_O_4_ nanoparticles observed in the size range of 7.4 nm to 45 nm [32]. A similar trend was observed in the case of Ni nanoparticles [34]. Figure 5a shows that coercivity depends on the size of the nanoparticles involved, and that for a series of MNPs over a range of sizes, MNPs go through two maxima in the 2 separate regimes (single-domain and multi-domain). Based on the coercivity values observed by Guardia *et al.*, we can conclude that the Fe_3_O_4_ MNPs synthesized by them are multi-domain MNPs above 17 nm. In the case of the Fe_3_O_4_ MNPs that are less than 20 nm in size, the presence of an oxidized layer of Fe_2_O_3_ on the surface of the Fe_3_O_4_ MNPs becomes significant, and the nanoparticles can no longer be classified as Fe_3_O_4_ MNPs [69]. We emphasize that this effect is in addition to the spin-disorder effect described earlier; consequently, the reduced Ms values might also arise from a higher ratio of low-magnetization maghemite (Fe_2_O_3_) to the high-magnetization magnetite (Fe_3_O_4_). Regardless of the composition, the size-dependence of MNP-properties is consistent. This phenomenon was demonstrated by Demortiere *et al.*, who studied the magnetic behavior of Fe_2.66_O_4_, a structure between Fe_3_O_4_ and Fe_2_O_3_, and observed that the saturation magnetization increased from 29 to 77 emu/g and blocking temperature increased from 10 to 100 K as the nanoparticle size was increased from 2.5 to 14 nm [70].

MNPs are also used in hyperthermia therapies, which involve increasing the temperature of an *in vivo* MNP-based therapeutic system to a level that either stimulates the immune system and potentiates other therapies (up to ~46 °C) or causes targeted ablation (above 46 °C) [71]. In this application, size becomes a critical tuning parameter since the application of an alternating current (AC) magnetic field will lead to heating that arises from either Neel or Brownian relaxation processes or hysteresis losses. Within the alternating magnetic field, either the magnetic moments rotate or the nanoparticle itself rotates, and when these MNPs relax back to their original magnetic field orientation (Neel relaxation time, *t*_N_, and Brownian relaxation time, *t*_B_, respectively), heat is released. The efficiency of heating for a magnetic material is described by the specific absorption rate (SAR), which is equal to the rate at which energy is absorbed per unit mass of the nanoparticles at a specific frequency [72] and is described as shown in Equation (4). Since the generation and absorption of heat arises from processes associated with relaxation and hysteresis losses, and since it is defined on a per-gram basis, it is also described as “specific loss power” (SLP) and is defined as:

(4) SAR (or SLP),W/g=C(ΔT/Δt)=(Area of the hysteresis loop)×(Frequency, f)

where C is the specific heat capacity of water, and ΔT/Δt is the rate of change of temperature. The SAR/SLP values that arise from the relaxation processes are roughly proportional to the Ms and magnetocrystalline anisotropy constant (K), and are inversely proportional to the size distribution of the nanoparticles [73]. In this review, we use both terms, SLP and SAR, to align with the nomenclature chosen by the respective authors to describe their results.

Mornet *et al.* showed that the SARs of MNPs were influenced by the composition, core diameter, coating, and frequency of the AC magnetic field [71]. In the case of large, ferromagnetic nanoparticles, heating occurs due to hysteresis losses and Brownian relaxation. For small nanoparticles in the single-domain or superparamagnetic range, hysteresis losses are negligible or absent, and heating arises from Neel and Brownian relaxation. The extent of the contribution of each mechanism is difficult to distinguish, but the dominant mechanism can be elucidated by determining the faster relaxation time [67]. In general, Neel relaxation dominates when nanoparticles are less than 20 nm, and Brownian relaxation dominates when the nanoparticles are larger than 20 nm [74]. Fortin *et al.* carried out a comprehensive study to distinguish between the contributions of Neel and Brownian relaxations to heat generation and SLP [75] as discussed in detail later in this section. A useful study by Jeun *et al.* established a threshold size (~9.8 nm) below which the measured SLP is insufficient for hyperthermia applications [76]. These researchers evaluated Fe_3_O_4_ MNPs of sizes in the range of 4.2 nm to 22.5 nm and determined that the SLP was insignificant (<45 W/g) at sizes <9.8 nm, but was greater by an order of magnitude in the size range of 11.8 to 22.5 nm. Lartigue *et al.* also observed a size threshold of 7 nm below which no significant heating was produced [77]. The SAR values jumped from almost zero for 4.1 and 6.7 nm MNPs to ~76 W/g for 35 nm rhamnose-coated Fe_3_O_4_ MNPs. In another study of magnetic nanoparticles having diameters of 5, 10, 12.8, and 14 nm, measurements at 400 kHz and 24.5 kA/m amplitude showed a maximum SLP of 447 W/g for the 14 nm Fe_3_O_4_ MNPs [78]. These data are important because a high SLP is necessary for efficient hyperthermia therapy with a minimal dose of MNPs in the body. Table 4 provides a summary of studies that have attempted to correlate size with SLP. A recent study presented and validated (with both commercial and in-house-synthesized Fe_3_O_4_ nanoparticles) an analytical model in which SLP is directly proportional to the AC magnetization for nanoparticles ranging in size from 5 to 600 nm; in contrast, there was no dependence on DC magnetization (Ms) [79].

To optimize the effectiveness of hyperthermia treatment using MNPs, Khandhar *et al.* tailored the nanoparticle size to the applied frequency [25]. Recent research indicates that SAR/SLP can be maximized if the total relaxation time matches the applied frequency [80], which along with the applied field has a FDA-regulated upper limit [79]. The total relaxation time is the sum of t_N_ and t_B_. Four equations correlate the relevant factors:

(5) $${\text{t}}_{\text{N}}={\text{t}}_{0}{\text{e}}^{\left(\text{KV}/\text{kT}\right)}$$

(6) $${\text{t}}_{\text{B}}=3\mu {\text{V}}_{\text{B}}/{\text{k}}_{\text{B}}\text{T}$$

(7) $$v_{\text{N}}=1/(2\pi \;{\text{t}}_{\text{N}})$$

(8) $$v_{\text{B}}=1/(2\pi \;{\text{t}}_{\text{B}})$$

where t_0_ is the relaxation time of non-interacting MNPs (~10^−9^ to 10^−12^ s), K is the anisotropy constant, k_B_ is the Boltzmann constant, V is the volume of nanoparticle, μ is the viscosity of the medium, V_B_ is the hydrodynamic volume, T is the temperature, ν_N_ is the frequency for maximum heating due to t_N_, and ν_B_ is the frequency for maximum heating due to t_B_.

Equipped with these equations, we can tailor the sizes of the nanoparticles for maximum heating. The above equations show how t_B_ depends directly on V_B_ and μ, and inversely on T; t_N_ varies exponentially with KV. We can also quantify the influence of size, viscosity of the suspension medium, the anisotropy constant, and temperature on the relaxation time and the heat output. Fortin *et al.* optimized the SLP by tuning the Brownian and the Neel relaxation times by varying the viscosity of the suspension medium (lower Brownian relaxation time for higher viscosity) and the size and composition of the nanoparticles (exponentially higher Neel relaxation time for MNPs with higher volume and higher anisotropy constants, which is a function of the MNP composition) [75]. The significant reduction in SLP for CoFe_2_O_4_ MNPs and the slight decrease in SLP for γ-Fe_2_O_3_ MNPs, when suspended in high-viscosity glycerol, confirmed the dominance of the Brownian and Neel relaxation time contributions for CoFe_2_O_4_ and γ-Fe_2_O_3_ MNPs, respectively. Below 10 nm, CoFe_2_O_4_ MNPs exhibited a higher SLP as compared to γ-Fe_2_O_3_ MNPs of the same size and appeared to be better candidates for hyperthermia applications.

As expected from Equation (1), the blocking temperature is generally found to be directly proportional to the nanoparticle volume/size. This relationship is in complete agreement with Monte Caro simulations demonstrating that the blocking temperature varied linearly with nanoparticle size [81]. Additionally the simulations also predicted a dependence of blocking temperature on the nanoparticle concentration, which has yet to be established experimentally. Rosenweig *et al.* computed the effect of different parameters on the heating rate of different superparamagnetic nanoparticles suspended in tetradecane when subjected to alternating DC currents [82]. In another simulation study, Carrey *et al.* evaluated the various theories describing relaxation losses and hysteresis losses [80] concluding that the anisotropy energy of the MNPs is the critical parameter to tune SAR. The authors also proposed a formula to estimate the optimum volume for the targeted anisotropy energy.

Barring a few examples, all of the nanoparticles in Table 3 exhibit blocking temperatures that are much lower than room temperature, which means that these nanoparticles are superparamagnetic at room temperature. Also, when the nanoparticles are small, the surface effects dominate (as expected from Equation (3)), giving rise to disordered spins of surface cations. Koseoglu *et al*. determined that the anisotropy constant stemming from this high anisotropy layer was inversely proportional to the size of Fe_3_O_4_ MNPs in the 1–11 nm range [83].

Given that magnetic behavior is strongly size-dependent, size can serve as a design parameter that can be readily manipulated to tune the magnetic properties of Ms, coercivity, blocking temperature, and SLP for increased efficiency in MNP applications. However, size manipulation alone might sometimes fail to produce the desired results.

### 3.2. Shape

As we have seen in the previous subsection, substantial efforts have been dedicated toward understanding the relationships between nanoparticle size and magnetic properties. In comparison, there is remarkably little research on the effect of shape on the magnetic properties of nanoparticles having the same volume or related size parameter. There are many studies on the synthesis of unique shapes of MNPs: for example, ferrite nanocubes [37,84], maghemite nanorods [85], NiFe nanowires [86], cobalt nanodiscs [39,87], magnetite tetrapods [88], and Au-MnO nanoflowers [89]. Table 5 lists studies that have compared various shapes and reported comparisons on the basis of their magnetic properties.

Among the properties evaluated, comparison of a set of CoFe_2_O_4_ cubes and spheres by Song *et al.* in 2004 found a large difference only in the coercivity [35]. The researchers attributed this difference to surface pinning that arises due to missing coordinating oxygen atoms. Unlike the curved topography in spherical CoFe_2_O_4_ MNPs, in the case of cubic CoFe_2_O_4_ MNPs, they hypothesized that fewer missing oxygen atoms, and thus less surface pinning, might have led to lower coercivity for the cubic structures. In two studies that compared cubic and spherical Fe_3_O_4_ MNPs, both Salazar-Alvarez *et al.* and Zhen *et al.* observed a higher blocking temperature for the spherical Fe_3_O_4_ MNPs [36,38]. Noh *et al.* corroborated this observation of high T_B_ for spherical nanoparticles in a comparison of cubic and spherical Zn_0.4_Fe_2.6_O_4_ MNPs [31]. These observations, and the explanation given above, are in accord with Equation 1; hence, the anisotropy constant for spherical nanoparticles is higher than cubic nanoparticles of the same volume.

Zhen *et al.* also observed that cubic MNPs had a higher Ms as compared to spherical MNPs of the same volume [38]. To explain the higher Ms in cubic nanoparticles as compared to spherical nanoparticles of the same volume, Noh *et al.* simulated the orientations of the magnetic spin structures in both a cube and a sphere using an object-oriented micromagnetic framework program (OOMMF) and found that, for their analysis, the disordered spins were 4% in cubic MNPs and 8% in spherical MNPs [31]. Based on these simulations, lower disordered spins in cubes should give rise to a higher Ms for cubic MNPs. However, a higher Ms for cubic nanoparticles as compared to spherical nanoparticles of the same volume appears not to be a universal observation. It becomes especially challenging to draw a correlation between shape and Ms for nanoparticles of dissimilar volumes in Table 5. A high Ms is expected for lower-volume nanoparticles due to its per-gram definition; however, a high Ms might be observed for a higher-volume cube due to lower disordered spins. Table 5 thus shows no unifying trend for any of the listed properties as a function of shape and volume. Likewise, since most of the shape-comparative studies have been performed for MNP sizes in the superparamagnetic regime or at least in the single-domain regime, it would be useful to the scientific community if future research focused on collecting magnetic data for varying shapes of nanoparticles spanning a larger range of sizes.

For the last couple of decades, a variety of MNPs have been evaluated for their use as contrast agents in magnetic resonance imaging (MRI). MRI is a powerful diagnostic technique in which a magnetic field is applied to a sample, and a magnetic dipole is induced in the nanoparticles used as contrast agents, which then affects the magnetic relaxation processes of the protons present in the surrounding fluid. On application of an external magnetic field, protons in the absence of MNPs experience a relaxation process that differs from than that observed in the presence of MNPs, and these processes occur along 2 pathways (longitudinal and transverse). The parameter T_2_ reflects the attenuation of the induced perpendicular magnetization, and T_1_ reflects attenuation back to the initial state. The decrease in the relaxation times (T_1_ or T_2_) under a local field variation (presence of MNPs) leads to enhanced image contrast. A reduction in T_1_ provides a positive contrast and a reduction in T_2_ provides a negative contrast. Thus, if MNPs accumulate in the tissue to be imaged, they can provide high-resolution MRI images. A recent review by Yoo *et al*. provides a more complete explanation [90]. An example would be the use of magnetic iron oxide nanoparticles as contrast agents to image the liver, spleen, and bone marrow due to their ability to reduce T_2_ in these tissues [91]. The contrast enhancement effects have been shown to be directly related to the Ms value of the nanoparticles [73]. Therefore, what is crucial in an MRI application is the relative strength of the magnetic field of the MNPs (indicated by their saturation magnetization) and their impact upon the spin-spin relaxation time (T_2_) of the surrounding protons.

The contrast-enhancing efficiency is described using relaxivity coefficients (r_1_, r_2_) [92,93], and these parameters are correlated using Equation (9):

(9) $$1/{\text{T}}_{i}=1/{{\text{T}}_{i}}^{0}+{\text{r}}_{i}\text{C }\;\;i=1,2$$

where T_1_, T_2_ are the longitudinal and transverse spin relaxation times in the presence of nanoparticles, T_1_^0^, T_2_^0^ are the relaxation times in pure water, r_1_, r_2_ are the relaxivity constants, and C is the concentration of the nanoparticles (contrast agent).

Experimentally, we can obtain r*_i_*_(_*_i_*_= 1,2)_ from a plot of 1/T*_i_*_(_*_i_*_= 1,2)_*versus* C. For example, in Equation (9), r_2_ is a constant, independent of concentration, and having a value associated with each contrast agent, reflecting the relative strength of the magnetic field surrounding the individual MNPs. To obtain enhanced negative contrast, T_2_ must be lowered, which requires either the use of an agent having a high r_2_ value or the use of a higher concentration of agent. Examples of agents with high r_2_ values are superparamagnetic nanoparticles with high saturation magnetization. To obtain enhanced positive contrast, T_1_ must be lowered, which requires the use of agents having a high r_1_ value. T_1_ agents to obtain enhanced positive contrast generally include gadolinium-based materials [93].

The use of superparamagnetic iron oxide nanoparticles as contrast agents has been approved clinically [2,94], and recent research involving a greater variety of superparamagnetic MNPs has been pursued, providing additional insight into the parameters that have an impact upon r_2_. Zhen *et al.* observed that, due to their higher crystallinity, cubic Fe_3_O_4_ MNPs showed four times smaller relaxation time and thus better image contrast when compared to spherical Fe_3_O_4_ MNPs [38]. On comparing faceted irregular (FI) CoFe_2_O_4_ with spherical CoFe_2_O_4_ MNPs, Joshi *et al.* observed a higher r_2_ (with respect to Ms) for the FI MNPs and a lower T_2_ [92]. In addition to the unique morphology-generated gradient for the magnetic field, the researchers attributed this variance to the higher surface-area-to-volume ratio for the FI MNPs as compared to the spherical MNPs, where more protons were present in the vicinity of this magnetic field, leading to faster relaxation (T_2_). The delivery of such nanoparticles to the tumor site takes place due to the combined phenomena of enhanced metabolism, permeation, and retention [95]. Large aggregates, however, may be eliminated from the body instead of accumulating in the tumor. The 22 nm (edge length) Fe_3_O_4_ nanocubes exhibited colloidal stability and a high r_2_ relaxivity, which enabled its successful use for *in vivo* MRI using a 3-T MR scanner [95].

We have noted that the SAR values increase with nanoparticle size. However, we have yet to establish the effect of the shape on SAR. Guardia *et al.* reported a maximum SAR of 2452 W/g Fe at 520 kHz and 29 kA/m for cubic Fe_3_O_4_ with an edge length of 19 ± 3 nm [72]. Additionally, Noh *et al.* reported a maximum SLP of 4060 W/g for larger-sized 40 nm (edge length) Zn_0.4_Fe_2.6_O_4_ nanocubes [31].

Based on the limited studies currently available in the literature, we can draw no broad conclusions in favor of a particular shape. However, MNPs with flat surfaces show promise for use in biomedical applications (e.g., biosensing, hyperthermia, and MRI), and warrant the pursuit of more shape-effect studies. Further, it is clear from the most recent MNP research that the impact of MNP shape on magnetic properties can be used as a powerful tool for modifying these properties to enhance the effectiveness of MNPs in a particular application.

### 3.3. Composition

Composition is the most commonly cited parameter responsible for determining the specific magnetic properties of a material. In the previous section, we classified all materials (without regard to their specific atomic content) based on their magnetic properties (*i.e.*, diamagnetic, paramagnetic, ferromagnetic, ferrimagnetic, and antiferromagnetic). These magnetic properties arise in the presence or absence of unpaired valence electrons located on the metal atoms or metal ions found in MNPs [96,97]. The orientation of the magnetic moment, μ, associated with the electrons defines the magnetic behavior. Using the magnetic moment of a single electron, 1.73 Bohr magnetons (BM), we can estimate the magnetic moment in a MNP. For example, with five unpaired electrons, Fe^+3^ has a moment of ~8.5 BM, which underlines the strong dependence of the composition (atomic state) on the magnetic behavior of a specific element. Additionally, the distribution of cations within the octahedral (O_h_) and tetrahedral sites (T_d_) of the commonly found spinel or inverse spinel crystal structures, is another critical determinant of μ. For example, in the crystal structure of Fe_3_O_4_ (which is actually FeFe_2_O_4_), Fe^+2^ and half of Fe^+3^ occupy octahedral sites, and the remaining half of the Fe^+3^ cation occupies a tetrahedral site in a face-centered cubic (*fcc*) lattice structure.

**Figure 6:**
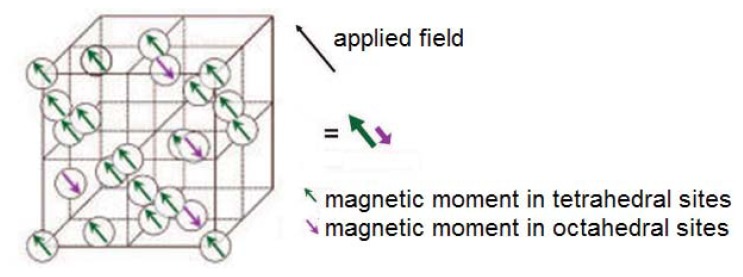
Tetrahedral and octahedral sites in an inverse spinel structure of ferrites. Reproduced with permission from [98].

As shown in Figure 6, the magnetic moments of the cations in the octahedral sites are aligned parallel to the magnetic field, and the ones in the tetrahedral sites are antiparallel, leading to a decrease in μ. Therefore, the net change in moment depends on the nature of the cations present in specific sites, such as the tetrahedral site for ferrites. Several research groups have investigated this structure by examining the effects of dopants (M cation) on the magnetic properties of ferrites (MFe_2_O_4_). The results from these studies are summarized of Table 6. Importantly, the properties of doped MNPs depend on the effectiveness of the synthetic procedure for consistently producing MNPs with crystal structures that are unvarying in their composition. Without a reliable basis for comparison, it can be challenging to compare the magnetic properties of MNPs synthesized by a variety of research groups using distinct synthetic routes. In Table 6, we highlight studies that have compared properties as a function of the relative ratio of cations, or the position and distribution of the cations, or otherwise systematically varying the composition of the MNPs.

The impact of the composition of the MNPs on magnetic properties has been studied by varying the precursor concentration, the method of synthesis, and the nature of the dopant, and by controlling post-synthetic cation exchanges. Based upon the presence of unpaired electrons, it is now possible to rationalize the magnetic behavior observed by Pereira *et al.* (Table 1) for Fe_3_O_4_ and MnFe_2_O_4_ MNPs as compared to CoFe_2_O_4_ [23]. As expected from the number of unpaired electrons for the substitutions made in these spinels, Deng *et al.* observed the highest magnetization for Fe_3_O_4_, but obtained a measurement that was anomalously low for MnFe_2_O_4_ [48]. In another study that compared MnFe_2_O_4_, FeFe_2_O_4_, CoFe_2_O_4_, and NiFe_2_O_4_ MNPs of the same 12-nm size, MnFe_2_O_4_ showed the highest magnetization [98]. The authors rationalized this result by comparing the crystal structure of each of the MNPs. The MnFe_2_O_4_ MNPs had a mixed spinel structure (Mn^+2^ and Fe^+3^ occupying both O_h_ and T_d_ sites), and the rest had an inverse spinel structure (Mn^+2^ and Fe^+3^ occupying O_h_ sites but only Fe^+3^ occupying the T_d_ sites).

Spinel ferrites have continued to be widely investigated, including recent detailed studies of the impact of cation placement on MNP magnetic field strength. Gabal *et al.* examined a series of Ni_0.8−_*_x_*Zn_0.2_Mg*_x_*Fe_2_O_4_ (*x* ≤ 0.8) ferrites and found that increasing the Mg^+2^ content during synthesis led to the replacement of the higher magnetic moment Ni^+2^ by the zero magnetic moment Mg^+2^, which led to decreases in the Ms and coercivity of the nanoparticles [99]. The same research group observed a similar reduction in the value of Ms when Ni^+2^ cations were replaced by Cu^+2^ cations in studies of MNPs having the form Ni_1−_*_x_*Cu*_x_*Fe_2_O_4_ (0 ≤ *x* ≤ 1) [43]. In addition to the nature of the cation itself, its relative distribution in the crystal structure is equally important, particularly in the case of spinel structures, where the distribution of cations in octahedral and tetrahedral sites defines the type of magnetic behavior. Turtelli *et al.* studied the magnetic properties of CoFe_2_O_4_ MNPs synthesized by sol-gel and ball milling methods and ascribed the difference in properties to dissimilar cation distributions formed during the two different synthetic methods [45].

While varying precursor ratios and synthesis methods offers one way of introducing a compositional change to MNPs, cationic exchange is another attractive technique for varying the cationic composition to tailor the magnetic properties of the resulting nanoparticles. Cationic exchange is especially attractive in the case of ferrite nanoparticles, where physical and magnetic properties can be tuned by replacing a cation without affecting its crystal structure [100]. Larumbe *et al.* studied the effect of nickel doping on Fe_3_O_4_ MNPs, where Ni^+2^ partially displaced Fe^+2^ from the octahedral sites [44]. Although there was no substantial change in the value of Ms, the blocking temperature for MNPs of the form Ni*_x_*Fe_3−_*_x_*O_4_ reached a maximum for Ni_0.06_Fe_2.94_O_4_ MNPs (*i.e.*, higher than that for Fe_3_O_4_ MNPs). As indicated by Equation 1, the blocking temperature is expected to vary linearly with volume, and the authors attributed the increase in blocking temperature to an increase in grain size. In addition to cationic exchange, Jang *et al.* demonstrated the importance of the proper replacement of Zn^+2^ dopants in Td sites [73] for optimum tuning of nanomagnetism. These authors observed a maxima in Ms at *x* = 0.4 for 15 nm Zn-doped nanoparticles of formula Zn*_x_*Mn_1−_*_x_*Fe_2_O_4_ and Zn*_x_*Fe_1−_*_x_*Fe_2_O_4_ that led to an eightfold to fourteenfold increase in MRI contrast and a fourfold enhancement in hyperthermic effects compared to conventional iron oxide nanoparticles. Furthermore, Fantechi *et al.* reported a detailed investigation on the effect of Co doping on 5 nm Co-doped nanoparticles, where Ms and K showed maximum values at intermediate compositions of 0.5 < *x* < 1 in Co_x_Fe_(8/3−2_*_x_*_/3)_O_4_ [100]. In another post-synthesis cationic exchange of Co^+2^ for Fe^+2^ in Fe_3_O_4_ (FeFe_2_O_4_) MNPs, the blocking temperature and the coercivity of the resulting CoFe_2_O_4_ MNPs increased significantly [47]; that is, the blocking temperature after Co^+2^ treatment of these 21-nm Fe_3_O_4_ MNPs increased to 310 K from 250 K, and the coercivity doubled. The authors suggested that a higher spin-orbit coupling at Co^+2^ sites led to an increased magnetic anisotropy and thus higher blocking temperature and coercivity. Cationic exchange is thus an effective tool for introducing alternative cations to produce various ferrite structures from Fe_3_O_4_ MNPs, to develop properties geared for particular applications.

In some cases, the magnetic behavior of the nanoparticles can depend on the solvent used during their synthesis. Clavel *et al.* observed that Mn-doped ZnO MNPs were paramagnetic from both solvent systems used (benzyl alcohol or anisole/benzyl alcohol at 95/5%); however, Co-doped MNPs were ferromagnetic when benzyl alcohol was used, and antiferromagnetic when the anisole/benzyl alcohol solvent system was used [101].

With only a few exceptions (e.g., Zn_0.4_Fe_2.6_O_4_ MNPs), alloyed MNPs such as FeCo generally exhibit higher Ms values (e.g., 150–200 emu/g) [102,103]. The enhancement has been attributed to the absence of the non-ferromagnetic “oxygen” component found in many of the alternative mixed-metal structures. Therefore, numerous recent studies focused on such alloy-based MNPs. For example, FeCo nanocubes of body diagonal 175, 350, and 450 nm synthesized by a liquid-phase reduction reaction showed an average Ms of 167 ± 4 emu/g [18]. Furthermore, a reductive thermal decomposition method employed by Chaubey *et al.* afforded FeCo spheres having 10 and 20 nm diameters with a size-dependent Ms of 129 emu/g and 207 emu/g, respectively [102]. These authors also found an optimum molar ratio of Fe:Co (1.5:1) for which the Ms was at a maximum. In a separate study of MNPs having the form Fe_100−x_Co_x_, Chinnasamy *et al.* also observed a higher Ms for Fe-rich nanoparticles as compared to Co-rich nanoparticles [42]. Rellinghaus *et al.* found that upon annealing, the face-centered tetragonal-(*fct*) structured FePt MNPs exhibited a high coercivity (5000–7000 kOe) [104]. The enhanced coercivity was attributed to the *fct* structure, while an also observed high blocking temperature was attributed to a high anisotropy constant, making FePt MNPs uniquely suitable for high-density data storage and hyperthermia applications [105]. Nanoparticles having the composition Fe_x_Pt_100−x_ (x = 70, 52, 48) synthesized by thermal decomposition and reduction exhibited blocking temperatures of 12 K for Fe_70_Pt_30_, 16.5 K for Fe_52_Pt_48_, and 30 K for Fe_48_Pt_52_ with diameters of 3.6, 3.1, and 3.8 nm, respectively. In evaluating the magnetic properties of FePt MNPs, Rellinghaus and co-workers examined how the difference in atomic volumes between Fe and Pt causes a distortion of the *fcc* structure when it transforms to the *fct* structure [104]. The distortion in symmetry of the Fe_x_Pt_100−x_ MNPs varies with the Fe:Pt ratio and is responsible for the variance in magnetocrystalline anisotropy as a function of composition. This variance in anisotropy then translates to the observed variance in blocking temperature in accord with Equation 1.

Another important MNP parameter that can be modulated by changes in composition is the Curie temperature (Tc), which is the temperature above which MNPs show zero magnetization. Overheating in hyperthermia applications can be avoided by using MNPs with a Curie temperature sufficiently low that they operate, not only as heating agents, but also as fuses [71]. During the past decade, several reports have focused on this aspect of “self-controlled” hyperthermia [106]. For example, when the aluminum content in MNPs having the formula Y_3_Fe_5−_*_x_*Al*_x_*O_12_ (0 ≤ *x* ≤ 2) was varied, the Curie temperature ranged from −40 to 280 °C. The composition was adjusted through cationic exchange, where the Fe^+3^ cations occupying the tetrahedral and octahedral sites were replaced by non-magnetic Al^+3^ cations, leading to a reduction in the saturation magnetization as the MNPs gained Al^+3^ content. The Tc for these MNPs reached room temperature when the Al^+3^ content was 1.5 < *x* < 1.8 [107]. A similar exchange of Sr^+2^ or Ti^+4^ in La_1−_*_x_*Sr*_x_*Mn_1−_*_y_*Ti*_y_*O_3_ MNPs led to a decrease in Tc from ~90 °C to ~20 °C; the Ti^+4^-substituted La_1−_*_x_*Sr*_x_*Mn_1−_*_y_*Ti*_y_*O_3_ MNPs had higher values of Ms and sharper Tc transitions when compared to the Sr^+2^-substituted La_1−_*_x_*Sr*_x_*Mn_1−_*_y_*Ti*_y_*O_3_ MNPs [108]. Another study by Miller *et al*. showed the importance of the phase of the material: variation in the composition of FeNi MNPs gave reduced values of Tc only for the γ-phase [109]. The Fe_73_Ni_27_ MNPs exhibited a Curie temperature of 550 °C in the bcc α-Fe phase and 120 °C in the γ-phase.

While composition provides an underlying definition of the magnetic behavior for these MNPs and directly affects the Ms and coercivity as shown in Table 6, the intrinsic phenomena that allows for the compositional tuning of MNPs to modulate Tc are not as well understood and require additional research. The strategy described above for applying compositional optimization to help restrict the upper heating limit for hyperthermia treatments might also lead to additional applications for remotely initiated self-regulated heating by MNPs.

### 3.4. Shell-Core Architecture

Nanoparticles are often coated with a selected material either (i) to make them biocompatible and stable in physiological fluids or (ii) to provide a modified surface that can be used for further functionalization; or (iii) to alter the magnetic properties of the core nanoparticle in a favorable manner [110,111]. The coating can be either non-magnetic or magnetic (antiferromagnetic, ferromagnetic, or ferrimagnetic) [112]. Regardless of the type of coating, there is usually some effect on the magnetic properties of the core. One effect is akin to the disordered spin layer that reduces the Ms of small nanoparticles (*vide supra*); since saturation magnetization is defined on a per gram basis, a non-magnetic coating (shell) will necessarily decrease its value. In the case of a magnetic coating, the core-shell interface interaction might lead to a change in anisotropy and a shift in the hysteresis loop. The shift of the hysteresis loop is “exchange bias” and it mainly arises due to interface coupling between two different types of layers (e.g., ferromagnetic and ferrimagnetic) [113,114]. The discussion that follows focuses on the impact of various types of coatings on the magnetic properties of surface-modified MNPs.

A coating of silica (SiO_2_) can transform a MNP by reducing problems associated with biocompatibility and offering the capacity to functionalize the surface of these nanomagnets [19]. Larumbe *et al.* evaluated the effect of SiO_2_ coating on Fe_3_O_4_ MNPs and found a reduced Ms and a lower coercivity, hence a lower specific absorption rate (SAR) for SiO_2_-coated Fe_3_O_4_ MNPs as compared to analogous uncoated Fe_3_O_4_ nanoparticles [21]. The authors attributed this decrease in both magnetization and SAR to surface spin effects. Moreover, they found that the blocking temperature was diminished for the SiO_2_-coated Fe_3_O_4_ MNPs. For thicker shells, the surface spin effects and the associated change in the anisotropy constant were accentuated and led to a further reduction in magnetization and SAR. Other silica-coated ferrite nanoparticles (MnFe_2_O_4_, CoFe_2_O_4_, NiFe_2_O_4_) showed similar results, with a reduced Ms after coating with silica; however, the decrease in the coercivity varied with the composition of the core [115,116]. For example, for the same size and coating, Vestal *et al.* showed that the Ms decreased as expected, but the coercivity decreased by 10% for silica-coated MnFe_2_O_4_ MNPs and 1% for silica-coated CoFe_2_O_4_ MNPs. This difference is likely due to the difference in magnetocrystalline anisotropy of MnFe_2_O_4_ (0.056 J/cm^3^) and CoFe_2_O_4_ (0.22 J/cm^3^). The change in anisotropy, and thus coercivity, is more marked in the case of composites with a lesser core anisotropy. In contrast to most studies that show a reduced magnetization for nanoparticles coated with a non-magnetic layer, Woo *et al.* demonstrated a higher Ms for silica-coated and amine-functionalized Fe_3_O_4_ MNPs [117]. Although some of the results obtained in core-shell MNP research might seem counterintuitive, it is clear from the results obtained from MNPs with a ferrite core that this aspect of MNP design is an important parameter that can be used to tailor the magnetic properties of the particles.

Noting the discussion above regarding hyperthermia, in the case of larger nanoparticles, a higher value of SAR (better MNP heating) depends on coercivity and Brownian losses. Consequently, although there may be no suitable alternative to coating an MNP system with a magnetization-reducing coating for a specific application, we can nevertheless choose the core-coating combination with the highest coercivity (composite with the lowest coercivity reduction after coating). Like silica coatings, a diamagnetic/nonmagnetic polymer layer offers similar advantages and disadvantages: enhanced biocompatibility and functionality but reduced magnetic properties. The effect of an N-isopropylacrylamide (NIPAM) coating on the magnetic properties of Fe_3_O_4_ MNPs is listed as an example in Table 7 [52].

In the subsection above describing the influence of shape on magnetic properties, we examined how diverse shapes affect the relaxation of the protons surrounding them, leading to changes in the imaging contrast. MNPs coated with water-stable and biocompatible materials have excellent qualities for MRI applications, and efforts to synthesize a broad variety of core-shell MNPs and to optimize their effectiveness as contrast agents are ongoing [118,119]. The past decade has seen numerous studies evaluating the effect of the core as well as the coating on the relaxation of the surrounding protons for their use as MRI contrast agents [120,121]. Although the magnetic core provides the field that alters the relaxation of the surrounding protons, the thickness and chemical composition of the coating influences the relative distance and general strength of the MNP magnetic field with regard to these protons. As the thickness of the coating (e.g., silica or polyethylene glycol) increases, the relaxivity (r_2_) decreases [122,123]. As we saw in the preceding subsection, core-shell composites that give reduced transverse relaxation times (T_2_) or increased relaxivities (r_2_) are more effective; therefore, the use of a thin coating will, in general, give a more effective contrast agent. However, in the case of silica-coated Fe_3_O_4_ MNPs, Ye *et al.* noted that, due to their permeability to water, the silica-coated Fe_3_O_4_ MNPs in their study exhibited a decreased longitudinal relaxivity (r_1_), leading to a net increase in the r_2_/r_1_ ratio, an indicator of MRI efficiency [123]. For this experiment, their silica-coated Fe_3_O_4_ nanocomposite was ~21 and ~14 times more efficient than the commercially available iron oxide contrast agents, Feridex and Resovist, respectively. Thus, the nature of the magnetic core, the composition of the coating (and its permeability and hydrophilicity), and the thickness of the coating can be used to enhance the efficiency of MNPs in MRI applications.

The influence of the shell on the magnetic properties is more interesting and provides us a higher tuning opportunity when both the core and the shell are magnetic, and also when the shell is metallic and the core is magnetic. Choo *et al.* observed an interesting interfacial effect at 20 K when hexagonal close packed (*hcp*) Ni nanoparticles that were antiferromagnetic below 12 K were coated with a *fcc* Ni shell that was superparamagnetic up to 360 K [49]. The magnetization peaked at this temperature regardless of the external magnetic field. In the case of Cu-capped (1.5 nm thick) Co nanoparticles, the surface anisotropy was higher than that for uncapped Co nanoparticle cores 1.1–4.5 nm in diameter [50]. When 2.7 nm Co nanoparticles were coated with varying thicknesses of Pt (up to 0.7 nm), the blocking temperature increased dramatically from 16 to 108 K [50]. The conclusion from this research is that capping MNP cores with a metal can increase the anisotropy and give a higher blocking temperature for core-shell MNPs. Such enhanced anisotropy characteristics have been attributed to the bonding of the d-orbital electrons of the core to the conduction band orbitals of the capping layer [50].

Enhancing the coercivity and remanent magnetization by exchange coupling a hard phase (high coercivity) with a soft phase (low coercivity) has been successfully used in multi-phase permanent magnets [126,127]. This basic idea, combined with pioneering research efforts, has paved the way for more recent studies focused on controlling the magnetic properties by varying the core-shell composition, shape, and dimensions. Zeng *et al.* synthesized an MNP designed with a hard FePt core (high coercivity) and a softer Fe_3_O_4_ shell (lower coercivity) and tuned the magnetic properties of the core-shell composite by varying the thickness of the shell [112]. Tailoring the magnetic properties by varying the thickness of the shell is experimentally simpler than modulating the MNP core phases [128]. For example, a prior study aimed at tuning the magnetic properties of MNPs was based on a combination of FePt and Fe_3_Pt in the core and required the separate syntheses of FePt and Fe_3_O_4_ MNPs followed by annealing a defined mixture of MNPs with precise control. The same research group demonstrated that tailoring the magnetic properties of MNPs could be accomplished by varying the composition of the shell [112]. The researchers examined both the softer-than-FePt Fe_3_O_4_ shell and the harder-than-FePt CoFe_2_O_4_ shell. In addition to a smooth hysteresis curve that demonstrated effective exchange coupling between the core and the shell, the coercivity varied inversely as the volume ratio of shell/core in the case of Fe_3_O_4_ shell/FePt core NMPs and varied inversely with the thickness of the CoFe_2_O_4_ shell for the other set of NMPs.

As noted above, Fe_3_O_4_ MNPs hold promise for their use in biomedical applications. However, if the standard Ms of ~100 emu/g for these MNPs can be further enhanced, they would find use in an even broader array of applications. Considering that iron metal has a higher magnetization than Fe_2_O_3_ or Fe_3_O_4_, it would appear that this element might also have significant potential for MNP applications. Unfortunately, iron is highly susceptible to oxidation, which severely limits the use of metallic Fe nanoparticles. However, Qiang *et al.* recently described the synthesis of a series of iron oxide-coated Fe core MNPs with coatings 2.5 nm thick and core diameters 2–100 nm; these oxidatively stable MNPs gave Ms values on the order of ~200 emu/g [129]. Furthermore, these unique MNPs are promising from an applications perspective because efficient and effective MRI contrast agents must have both high magnetization and elements that enhance the relaxation times of the protons in the surrounding environment [22]. Importantly, these Fe core-iron oxide shell nanoparticles, which consist of α-Fe at the core and γ-Fe_2_O_3_ or Fe_3_O_4_ as the shell, possess both characteristics [20].

We also noted in a preceding subsection that exchange bias (measured as a shifting of the hysteresis curve) occurs in the coupling of ferromagnetic and ferrimagnetic layers; this bias can also exist in ferrimagnetic layers and disordered spin layers [53]. Ong *et al.* compared Fe-Fe_3_O_4_ core-shell MNPs and Fe_3_O_4_ hollow-shell MNPs and found that because of interfacial spin interactions, there was a much higher exchange bias (1190 Oe) in the Fe-Fe_3_O_4_ core-shell MNP as compared to that observed in the hollow-shell MNP (133 Oe) [53]. In the case of hollow-shell MNPs, the broken exchange bonds on the inner surface induced a surface-spin disorder, giving a core-shell structure of disordered spins and Fe_3_O_4_ shell. Their studies demonstrated that the effect of the interfacial spin interactions was amplified in the case of a ferromagnetic core and ferrimagnetic shell when compared to a disordered spin core and Fe_3_O_4_ shell (ferrimagnetic shell alone). In contrast, Khurshid *et al.* reported an approximately 7-fold enhancement of exchange bias (~96 mT) in 18.7 nm hollow maghemite nanoparticles as compared to that (~17 mT) observed in 18.5 nm solid γ-Fe_2_O_3_ [130]. Additionally, the researchers attributed the higher T_B_ for the hollow γ-Fe_2_O_3_ (as compared to solid γ-Fe_2_O_3_) to the spin disorder enhancing the surface anisotropy. This increase in surface anisotropy leading to higher blocking temperatures for hollow nanoparticles has been also illustrated for NiFe_2_O_4_ MNPs (solid Ni_33_Fe_67_ core/NiFe_2_O_4_ shell and NiFe_2_O_4_ shell only) [131]. These studies highlight the importance of surface spin disorder, and that hollow nanoparticles provide another tool for tuning magnetic properties.

The interplay of the saturation magnetization, coercivity, magnetic anisotropy energy barrier (reflected in the anisotropy constant, K), and viscosity of the suspension medium is critical for MRI and hyperthermia applications [61]. Since the anisotropy constant reflects an intrinsic property of the material used to produce the nanoparticle, composition is also a known parameter that can be used to tune the SAR/SLP. However, as further discussed in this section, tuning K by varying the composition is challenging, and an exchange-coupled magnet has proven more effective for developing tunable magnetic properties and optimizing application efficiency. For hyperthermia applications, an SAR of 1 kW/g is necessary at 100 kHz and 20 mT (human-compatible conditions). Meffre *et al.* have reported a high SAR of 415 W/g at 96 kHz and 20 mT for 13.6 nm iron carbide@iron nanoparticles [132]. After confirming the presence of exchange-coupling between the shell and the core of their core-shell nanoparticles by a smooth hysteresis curve, Lee *et al.* [125] demonstrated that their composite particles exhibited a significant enhancement in SLP (1000 to 4000 W/g) as compared to single-component MNPs (100 to 450 W/g) and commercial Ferridex nanoparticles (115 W/g).

A variety of distinct combinations for the assembly of core-shell MNPs continue to be synthesized and characterized; these studies highlight the experimental capacity to optimize magnetic properties such as magnetization and coercivity by fine-tuning the composition and thickness of the core-shell architectures. Some of these studies are summarized in Table 7. Of particular interest, Noh *et al.* synthesized cubes of CoFe_2_O_4_-coated Zn_0.4_Fe_2.6_O_4_ cores and observed a smooth hysteresis curve and a 14-fold increase in coercivity as compared to the core alone [31]. This increase translated into a dramatically higher SAR for the shell-core MNPs (10,600 W/g) when compared to that of MNPs composed of just the core (4060 W/g).

As discussed earlier, a “domain wall” separates the domains, and its thickness depends on the anisotropy of the material. Recent reports have noted that the transition between the hard and soft phases is most effective (*i.e.*, characterized by a smooth transition curve) when the shell thickness is about twice the width of the domain wall (e.g., ~20 nm) [31,128]. A single, smooth hysteresis curve for a multi-layered nanocomposite system is thus an indication of a near-perfect coupling at the interface. As noted above, for MNP interactions within an alternating magnetic field, magnetic nanoparticles store and dissipate energy via t_N_, t_B_, and hysteresis losses. The shell-core architecture (composition and dimensions) therefore provides yet another avenue for maximizing coercivity (and thus SAR/SLP), providing a route for developing even more effective hyperthermia treatments.

In this review, we have focused on the physical parameters that offer opportunities for tuning and optimizing the magnetic behavior of MNPs. Importantly, there are additional strategies that harness the collective properties of nanoparticles [133], including the effects of multi-core assembly [134], concentration/dipolar interactions [135], and clustering [133,136]. An adequate description of these efforts warrants a separate review.

## 4. Conclusions

The various studies summarized in this brief review collectively illustrate the challenges facing efforts to provide a single algorithm for optimizing the properties of MNPs for selected applications. The broad dependence of the magnetic properties on multiple interlinked factors is especially daunting. Nevertheless, from this complex network of parameters, we have sorted and highlighted several important correlations between certain magnetic properties of MNPs (saturation magnetization, coercivity, blocking temperature, and relaxation time) to selected physical parameters (size, shape, composition, and shell-core architecture) that can be selectively and judiciously modulated. The goal of this review has been to provide greater access to this array of research and encourage the use of these parameters to enhance the specific properties of MNPs. Such nanoparticle modulation should lead to an even wider range of applications for this interesting class of nanomaterials.

## Figures and Tables

**Table 1:** Tunable magnetic properties important for biomedical applications.

Tunable property	Application
Saturation magnetization (Ms)	Biosensing [5], Drug Delivery [7,8], Magnetic Resonance Imaging (MRI) [22]
Coercivity (Hc)	Biosensing [5], Hyperthermia [9]
Blocking temperature (T_B_)	Biosensing, Drug Delivery [7,8], Hyperthermia [9]
Neel and Brownian relaxation time of nanoparticles (t_N_ & t_B_)	Biosensing [5], Hyperthermia [9]

**Table 2:** Parameters influencing tunable magnetic properties.

Influencing parameters	Partial list of references
Size	[23–30]
Shape	[31–40]
Composition (changing elements, doping, changing cation distribution in the crystal)	[41–48]
Shell-core design	[49–54]

**Table 3:** Magnetic properties of a variety of types of MNPs of varying sizes.

Reference	MNP	Size (nm)	Ms (emu/g)	Coercivity (G)	T_B_ (K) or neel temperature (T_N_, K)
Caruntu *et al.* [24]	Fe_3_O_4_	6.6	71	16	203
		11.6	77	15	264
		17.8	83	3	>300

Peddis *et al.* [26]	CoFe_2_O_4_	2.8	109	-	51
		2.9	89	-	80
		6.7	78	-	126

Guardia *et al.* [32]	Fe_3_O_4_	4.2	75	318	19
		7.4	70	270	28
		8.1	65	70	49
		17	82	364	>275
		45	92	340	>275

Han *et al.* [27]	HoMnO_3_	30	0.3 (5K)	382	50 (T_N_)
		200	0.1 (5K)	~0	70 (T_N_)

Pereira *et al.* [23]	Fe_3_O_4_	4.9	60.4		33.9
		6.3	64.8		56.2
		8.6	58.0		96.0

	CoFe_2_O_4_	4.2	30.6		89.4
		4.8	46.0		149.2
		18.6	48.8		286.4

	MnFe_2_O_4_	9.3	57.1		397.7
		11.7	54.6		91.0
		59.5	35.2		96.6

He *et al.* [34]	Ni	24	25.3	120	-
		50	32.3	79	-
		96	40.6	18	-
		165	46.7	146	-
		200	52.0	158	-

Noh *et al.* [31]	Zn_0.4_Fe_2.6_O_4_	18	165	60	320
		60	190	140	-
		120	200	60	-

**Table 4:** SAR/SLP of MNPs of varying sizes.

Reference	MNP	Size (nm)	SAR/SLP (W/g)	Frequency/amplitude
Mornet *et al.* [71]	Single-domain Fe_3_O_4_ coated with dextran	10–12	210	At 880 kHz and 7.2 kA/m
	Single-domain Fe_3_O_4_ coated with carboxymethyl dextran	6–12	90	At 880 kHz and 7.2 kA/m
	Multi-domain Fe_3_O_4_	150–200	45	At 880 kHz and 7.2 kA/m
	Single-domain Fe_3_O_4_	8	21	At 300 kHz and 6.5 kA/m
	Single-domain γ-Fe_2_O_3_	5	524	At 500 kHz and 12.5 kA/m
	Single-domain γ-Fe_2_O_3_	7	626	At 500 kHz and 12.5 kA/m

Jeun *et al.* [76]	Fe_3_O_4_	4.2	45	At 110 kHz and 140 Oe
		5.8	30	
		7.9	28	
		9.8	28	
		11.8	150	
		14.0	201	
		16.5	249	
		20.0	309	
		22.5	322	

Mueller *et al.* [30]	Fe_3_O_4_	10.9	216	At 210 kHz and 30 kA/m
		15.2	702	

Fortin *et al.* [75]	γ-Fe_2_O_3_	5.3	4	At 700 kHZ and 24.8 kA/m
		6.7	14	
		8.0	37	
		10.2	275	
		16.5	1650	
	CoFe_2_O_4_	3.9	40	
		9.1	360	
	γ-Fe_2_O_3_ in 95% water 5% glycerol	7.1	135	
	γ-Fe_2_O_3_ in 40% water 60% glycerol	7.1	125	
	γ-Fe_2_O_3_ in 0% water 100% glycerol	7.1	100	
	CoFe_2_O_4_ in 95% water 5% glycerol	9.7	420	
	CoFe_2_O_4_ in 40% water 60% glycerol	9.7	145	
	CoFe_2_O_4_ in 0% water 100% glycerol	9.7	90	

Lartigue *et al.* [77]	Fe_3_O_4_ coated with rhamnose	4.1	0	At 168 kHz and 21 kA/m
		6.7	0	
		10.0	30	
		16.2	61	
		35.2	76	

**Table 5:** Comparison of magnetic properties of various shapes of nanoparticles.

Reference	MNP	Shape	Size (nm) volume comparison	Ms (emu/g)	Coercivity	T_B_ (K)
Song *et al.* [35]	CoFe_2_O_4_	Sphere	10	80	16000 Oe	275
		Cube	8	80	9500 Oe	275
			V_sphere_ = V_cube_			

Salazar-Alvarez *et al.* [36]	γFe_2_O_3_	Sphere	14.5	75	30 mT	235
		Cube	12 Side	75	33 mT	190
			V_sphere_ = V_cube_			

Chou *et al.* [40]	FePt	Cube	11.8	2.5	164 Oe	50
		Octapod	12 body dia	2.0	1461 Oe	95
		Cuboctahedron	6.8 dia	0.1	11 Oe	20
			V_cube_ > V_octapod_ > V_octahedron_			

Zhen *et al.* [38]	Fe_3_O_4_	Cube	8.0 Side	40	0	60
		Sphere	8.5	31	0	100
			V_cube_ > V_sphere_			

Montferrand *et al.* [33]	Fe_3_O_4_ (includes γFe_2_O_3_)	Cube	12 Side	40	0	
		Rod	12 Width	18	4.4 kA/m	
		Sphere	12	80	0	
		Octahedron	12 Width	80	0	
			V_cube_ > V_rod_ > V_sphere_ > V_octahedron_			

Noh *et al.* [31]	Zn_0.4_Fe_2.6_O_4_	Sphere	22	145	0	360
		Cube	18	165	0	320
			V_sphere_ = V_cube_			

* G = 10^−1^ mT, G = (1/4π) kA/m.

**Table 6:** Effect of composition on magnetic properties.

Reference	Nanoparticle	Size (nm)	Method of changing composition	Ms (emu/g)	Coercivity
Deng *et al.* [48]	FeFe_2_O_4_	200	Varying precursors	81.9	
	MnFe_2_O_4_	200		53.2	
	CoFe_2_O_4_	200		61.6	
	ZnFe_2_O_4_	200		60.0	

Lee *et al.* [98]	FeFe_2_O_4_	12	Varying precursors	101	
	MnFe_2_O_4_	12		110	
	CoFe_2_O_4_	12		99	
	NiFe_2_O_4_	12		85	

Gabal *et al.* [99]	Ni_0.8−_*_x_*Zn_0.2_Mg*_x_*Fe_2_O_4_		Varying precursor ratios		
	*x* = 0	36		43.1	65.8 G
	0.2	41		41.7	57.0 G
	0.4	45		41.0	35.0 Gm
	0.6	35		30.4	17.4 G
	0.8	59		36.1	11.9 G

Larumbe *et al.* [44]	FeFe_2_O_4_	8	Varying precursor ratios	80.1	153 Oe
	Ni_0.04_Fe_2.96_O_4_	8		84.2	180 Oe
	Ni_0.06_Fe_2.94_O_4_	10		80.5	250 Oe
	Ni_0.11_Fe_0.89_O_4_	8		82.8	190 Oe

Turtelli *et al.* [45]	CoFe_2_O_4_–different		Varying synthesis methods		
	cation distribution				
	Ball milling	200		80.9	1750 Oe
	Sol gel	200		83.1	500 Oe

**Table 7:** Influence of various types of coatings on the properties of magnetic nanoparticles.

Reference	Nanoparticle (Shell@Core)	Size (nm)	Ms (emu/g)	Coercivity (Oe)	T_B_ (K)	SAR/SLP (W/g)
Larumbe *et al.* [21]	Fe_3_O_4_	5	72	0	160	1.5
	SiO_2_@Fe_3_O_4_	7.5	37	0	120	1.08
						At 340 kHz and 170–340 Oe

Shamim *et al.* [52]	Fe_3_O_4_	9.3	75.7	1.1		
	PNIPAM@Fe_3_O_4_	12	51.6	5		

Ebbing *et al.* [46]	Co	2.7			16	
	Pt@Co	3.0			108	

Yang *et al.* [124]	MnFe_2_O_4_	202	74	89		
	Ni@MnFe_2_O_4_	200	30	89		

Zeng *et al.* [54]	FePt	4	1040	5500		
	Fe_3_O_4_@FePt	6	950	13500		

Lee *et al.* [125]	CoFe_2_O_4_	9				450
	MnFe_2_O_4_	15				450
	MnFe_2_O_4_@CoFe_2_O_4_	12				2250
						At 500 kHz and 37.3 kA/m

Noh *et al.* [31]	Zn_0.4_Fe_2.6_O_4_	50	190	140	320	4060
	CoFe_2_O_4_shell@Zn_0.4_Fe_2.6_O_4_	60	130	1900		10600
						At 500 kHz and 37.4 kA/m
